# Robust Low-Overlap Point Cloud Registration via Displacement-Corrected Geometric Consistency for Enhanced 3D Sensing

**DOI:** 10.3390/s25144332

**Published:** 2025-07-11

**Authors:** Xin Wang, Qingguang Li

**Affiliations:** School of Computer, Electronics and Information, Guangxi University, Nanning 530004, China; wx__fm@163.com

**Keywords:** point-cloud registration, low-overlap sensor data, geometric consistency, predictive displacement rectification, LiDAR, sensor data fusion, 3D sensing

## Abstract

Accurate alignment of 3D point clouds, achieved by ubiquitous sensors such as LiDAR and depth cameras, is critical for enhancing perception capabilities in robotics, autonomous navigation, and environmental reconstruction. However, low-overlap scenarios—common due to limited sensor field-of-view or occlusions—severely degrade registration robustness and sensing reliability. To address this challenge, this paper proposes a novel geometric consistency optimization and rectification deep learning network named GeoCORNet. By synergistically designing a geometric consistency enhancement module, a bidirectional cross-attention mechanism, a predictive displacement rectification strategy, and joint optimization of overlap loss with displacement loss, GeoCORNet significantly improves registration accuracy and robustness in complex scenarios. The Attentive Cross-Consistency module of GeoCORNet integrates distance and angular consistency constraints with bidirectional cross-attention to significantly suppress noise from non-overlapping regions while reinforcing geometric coherence in overlapping areas. The predictive displacement rectification strategy dynamically rectifies erroneous correspondences through predicted 3D displacements instead of discarding them, maximizing the utility of sparse sensor data. Furthermore, a novel displacement loss function was developed to effectively constrain the geometric distribution of corrected point-pairs. Experimental results demonstrate that our method outperformed existing approaches in the aspects of registration recall, rotation error, and algorithm robustness under low-overlap conditions. These advances establish a new paradigm for robust 3D sensing in real-world applications where partial sensor data is prevalent.

## 1. Introduction

The rapid proliferation of 3D sensing technologies—spanning LiDAR, depth cameras, and structured-light sensors—has revolutionized perception capabilities in robotics, autonomous navigation, and environmental monitoring [1]. Central to these applications is point cloud registration, a fundamental process that aligns partial scans from disparate sensor viewpoints into a unified 3D representation [2]. However, in real-world sensing scenarios characterized by occlusions, limited sensor field-of-view, or dynamic obstacles, overlapping regions between point clouds frequently fall below 30%. This low-overlap regime severely undermines registration robustness, inducing alignment failures that propagate errors to downstream perception tasks such as simultaneous localization and mapping (SLAM) or 3D reconstruction. Current mainstream methods typically first construct a correspondence matrix and then decompose it to solve for optimal rigid transformation parameters [3].

Traditional methods of point cloud registration primarily employ optimization-based techniques to determine the optimal rigid transformation matrix between two point clouds [4,5]. As this represents a nonlinear problem, it remains highly challenging. The core methodology involves two components: robust correspondence estimation and precise transformation estimation. Correspondence estimation identifies corresponding point-pairs between source and target point clouds, while transformation estimation utilizes these correspondences to compute the rigid transformation matrix. These two stages iteratively refine the transformation until convergence. Widely adopted methods include iterative closest points (ICP) [6] and its variants [7,8], which implement diverse correspondence search strategies. However, these approaches require complex heuristics to address noise, outliers, density variations, and partial overlaps, thereby increasing computational costs. Furthermore, traditional methods often rely on handcrafted feature descriptors, which limits their generalizability and makes them perform poorly in low-overlap scenarios [9].

In recent years, deep learning has gained prominence in computer vision, and numerous learning-based point cloud registration methods have emerged [10,11]. These approaches demonstrate the ability to handle large rotational angles and generally outperform traditional global registration methods in speed. Unlike conventional optimization-based methods, deep learning-based registration approaches fall into two categories: feature learning-based methods and end-to-end learning-based methods.

Feature learning-based methods focus on using deep neural networks to extract discriminative features for robust correspondence estimation [12]. Specifically, deep neural networks learn robust feature correspondences, followed by one-step transformation estimation (e.g., via RANSAC [13]). While these methods achieve high efficiency and accuracy in high-overlap scenarios by effectively obtaining sufficient matches and stable geometric structures, they remain vulnerable to noises and outliers. Insufficient geometric modeling may also lead to computational redundancy, as global features often fail to capture fine details [14]. In low-overlap scenarios, the limitations of global features in capturing local geometric details exacerbate structural modeling deficiencies. Additionally, the reliability of initial matches becomes particularly problematic in such cases: limited overlapping regions induce bias in global feature-based correspondences, degrading initial match quality [15]. Meanwhile, existing feature learning-based methods of point cloud registration employ a direct outlier removal strategy, resulting in underutilization of outlier information and further compromising registration robustness.

End-to-end learning-based registration methods attempt to solve registration problems through end-to-end neural network optimization [16]. Unlike the aforementioned feature-learning approaches, end-to-end methods embed transformation estimation into neural network optimization. In high-overlap scenarios, end-to-end methods leverage abundant correspondences for precise registration but exhibit noise sensitivity and high computational complexity due to insufficient geometric modeling. In low-overlap scenarios, such methods face multiple challenges despite attempts to enhance local features and iteratively refine pose estimation [17]. First, while local feature enhancement aims to improve geometric modeling, the absence of explicit structural constraints leaves these methods susceptible to noise interference [18]. Second, sparse correspondences amplify the issue of unreliable initial matches, causing iterative optimization to converge to local minima [19]. Finally, existing end-to-end methods discard outlier pairs via hard thresholds, failing to exploit potential geometric information in correspondences and aggravating information loss in scenarios of low overlap.

In summary, robust alignment of 3D point clouds acquired by ubiquitous sensors (e.g., LiDAR, depth cameras) is paramount for enhancing perception capabilities in robotics, autonomous navigation, and environmental reconstruction. However, the inherent limitations of real-world sensing scenarios like occlusions, restricted sensor field-of-view, and dynamic obstacles frequently lead to severely low-overlap data (overlap < 30%). This low-overlap regime poses significant challenges to traditional and learning-based registration methods, primarily stemming from the following: (1) inadequate exploitation of the inherent geometric constraints imposed by rigid sensor motion on the data; (2) unreliable initial feature matching due to sparse and noisy sensor data; and (3) inefficient utilization of valuable but erroneous sensor correspondences (outliers), often discarded by conventional strategies, further exacerbating data sparsity. To overcome these critical bottlenecks for robust 3D sensing, we propose GeoCORNet, a novel deep neural network. GeoCORNet integrates synergistic innovations designed specifically to enhance the reliability of registration from low-overlap, real-world sensor data, achieving significant improvements in accuracy and robustness through geometric consistency enhancement, intelligent outlier rectification, and joint optimization.

Our main contributions are summarized as follows:

1. Dual-Consensus Attentive Feature Enhancer (DCA): This module improves the spatial consistency constraints in SC2-PCR [20] by incorporating angular consistency through cosine similarity. DCA module enforces geometric constraints on matched point-pairs by jointly considering spatial distance and directional consistency, substantially improving matching robustness in complex scenes.
2. Bidirectional Cross-Attention for Sensor Data Fusion: Unlike unidirectional attention, our novel bidirectional mechanism enables symmetric feature interaction between source and target point clouds (representing distinct sensor viewpoints). This mutual attention dynamically focuses on salient overlapping regions critical for alignment while actively suppressing interference from non-overlapping or noisy sensor data, thereby providing a significantly more reliable set of initial correspondences for downstream optimization.
3. Predictive Outlier Correction: Departing fundamentally from the prevalent paradigm of outlier removal, we propose a novel displacement prediction-based correction strategy. Recognizing the value of potentially informative but misaligned sensor measurements in low-overlap scenarios, we employ a lightweight MLP to predict 3D displacement offsets for both points in outlier pairs. This intelligent rectification transforms erroneous correspondences into geometrically plausible ones, maximizing the utility of sparse sensor data and enabling more accurate global transformation estimation, leading to more efficient and precise registration.
4. Displacement Loss Function: To ensure the corrected correspondences conform to the expected geometric structure of the sensor data, we design a novel displacement loss function. This loss explicitly constrains the geometric distribution of the rectified outlier point-pairs to align with that of the inliers, guaranteeing the effectiveness of the predictive correction process and enhancing overall registration robustness.
5. Experimental results on the public dataset 3DMatch and the low-overlap dataset 3DLoMatch demonstrate that our method effectively addresses the registration of low-overlap point cloud, confirming its effectiveness and generalizability. The proposed approach surpasses existing methods in registration recall rate, rotation error, and algorithmic robustness.

## 2. Related Works

### 2.1. Feature Learning-Based Point Cloud Registration

Feature learning-based point cloud registration methods leverage deep learning techniques to extract point cloud feature representations and establish correspondences, achieving efficient and robust registration.

In general scenarios, PointNet [21] laid the foundation for subsequent research by extracting global features via shared MLPs and symmetric functions. However, its inability to capture local geometric features limited its performance on complex structures. Subsequently, PointNet++ [22] extended this framework through hierarchical feature learning and local region sampling, significantly enhancing local geometric feature extraction. While this improved feature representations for registration, it remained constrained in low-overlap and rotation-variant scenarios. RIGA [12] achieved efficient registration via local geometric feature extraction and global optimization but exhibited sensitivity to noise and partial overlaps, heavily relying on initial match quality. D3Feat [23] and FCGF [24] advanced point cloud feature extraction through joint keypoint detection as well as feature learning and sparse convolutions, respectively. D3Feat improved registration accuracy by co-learning keypoints and descriptors, while FCGF enhanced efficiency via dense feature extraction. SpinNet [25] introduced rotation-equivariant convolutions to address rotational sensitivity, further boosting feature matching robustness. In low-overlap scenarios, Predator [26] dynamically filtered overlapping regions with attention mechanism but struggled with symmetric structures and feature discriminability. GeoTransformer [14] combined Transformer-based global modeling with geometric invariance for rigid transformation invariance, albeit with high memory costs for large-scale point clouds.

Our method inherits the core concept of geometric-invariant feature learning but uniquely integrates angular consistency to construct dual-dimensional geometric constraints, substantially mitigating mismatches caused by symmetric structures or repetitive local features.

### 2.2. End-to-End Learning-Based Point Cloud Registration

End-to-end learning-based methods jointly optimize feature extraction and correspondence estimation, and they pose refinement within a unified deep learning framework, reducing manual intervention while improving overall accuracy and robustness.

In general scenarios, PointNetLK [16] combined PointNet [21] with the Lucas–Kanade algorithm [27] for end-to-end pose regression but suffered from sensitivity to initial poses and frequent failures in low-overlap cases. Based on this, Deep Closest Point (DCP) [17] implicitly learned feature correspondences via deep networks and solved transformations with SVD, though its performance degraded in low-overlap settings due to dependency on accurate feature matching. 3DRegNet [28] bypassed initial pose requirements by directly regressing transformation matrices but showed limited robustness in low-overlap scenarios. For partial overlaps, RPM-Net [18] enhanced robustness with differentiable soft correspondence matching and iterative refinement, while DGR [29] achieved outperformance via a differentiable global registration framework integrating 6D convolutional networks and weighted Procrustes optimization. HRegNet [30] adopted a hierarchical coarse-to-fine strategy for complex and low-overlap scenarios but struggled with noise and outliers. OMNet [19] learned overlap masks to filter non-overlapping points but faced generalization limitations due to training data biases. Despite efficiency on benchmarks like 3DMatch and KITTI, these methods still grappled with extreme low-overlap (<10%) adaptability, high computational costs for large-scale data, noise sensitivity, and initial pose dependency.

Unlike black-box regression in existing end-to-end approaches, our method explicitly models geometric constraints, achieving superior robustness in low-overlap, noisy, and initial pose-deviated scenarios.

### 2.3. Outlier Rejection for Point Cloud Registration

Due to the challenges such as partial overlaps or feature ambiguity, correspondence estimation tends to generate outliers. Thus, outlier rejection becomes particularly critical when the overlapping regions are sparse.

PointDSC [31] introduced a spatial consistency score to filter outliers, significantly improving low-overlap robustness. SC2-PCR [20] introduced a hierarchical matching strategy to further enhance registration accuracy. The method proposed a two-stage registration framework based on superpoint matching. During the coarse registration phase, superpoint matching reduced the impact of outliers; while in the fine registration phase, correspondences were further optimized. This coarse-to-fine strategy progressively refined the registration results. QGORE [32] proposed a quadratic-time outlier rejection technique to enhance performance. PointTr [33] leveraged Transformer-based global–local feature interaction to suppress outliers, and 3DPCP-Net [34] incorporated normal vector consistency and progressive guidance for reliable correspondence selection. These methods share a common strategy: filtering initial matches via spatial or normal consistency or deep features to remove outliers. However, direct outlier removal risks discarding valuable information, particularly in low-overlap scenarios where correspondences are already scarce.

Different from conventional outlier rejection, our method proposes a displacement prediction-based correction strategy. We first filter obvious outliers and then rectify remaining ones via displacement prediction, balancing information retention and computational efficiency for low-overlap, large-scale registration.

## 3. Methods

Given a source point cloud $P=\{p_{i}\in R^{3}|i=1,2,\ldots ,N\}$ and a target point cloud $Q=\{q_{i}\in R^{3}|i=1,2,\ldots ,M\}$, where N and M denote the number of points in P and Q, respectively, GeoCORNet aims to predict the optimal rotation matrix R and translation vector t from P to Q in low-overlap scenarios.

**Figure 1:**
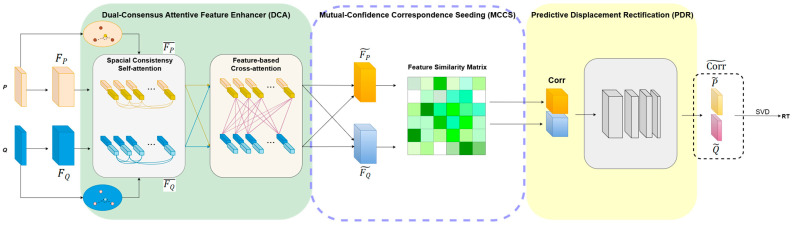
The architecture of GeoCORNet. The network takes the coordinates of the source point cloud P and target point cloud Q as input and outputs the rigid transformation RT=[R,t]. The framework has three core components. Dual-Consensus Attentive Feature Enhancer (DCA) module utilizes geometric consistency constraints and bidirectional cross-attention to aggregate weighted features, generating enhanced features $\tilde{F_{P}}$ and $\tilde{F_{Q}}$. Based on the enhanced feature similarity, Mutual-Confidence Correspondence Seeding (MCCS) module builds high-confidence seed correspondence set Corr. Predictive Displacement Rectification (PDR) module rectify the outlier offsets in Corr, producing an optimized correspondence set $\tilde{Corr}$. Finally, the rigid transformation RT is derived via Singular Value Decomposition.

As illustrated in Figure 1, for the given source point cloud P and target point cloud Q, the framework first extracts highly robust fused features $\tilde{F_{P}}$ and $\tilde{F_{Q}}$, integrating spatial geometric and cross-cloud contextual information via the DCA module. The Mutual-Confidence Correspondence Seeding (MCCS) module then picks out the top-k initial correspondences to construct a seed correspondence set Corr={($p_{i},q_{i}$)$\in R^{k\times 6}|i=1,2,\ldots ,k$}, which exhibit relatively reliable matches despite still existing potential outliers. Each seed correspondence point-pair in Corr is represented as ($p_{i},q_{i}$)$\in R^{k\times 6}$, where $p_{i}\in P,q_{i}\in Q$.

Assuming that a coarse transformation derived from Corr approximately aligns P and Q, the Predictive Displacement Rectification (PDR) module further refines the positional offsets of outlier point-pairs in Corr, yielding an optimized correspondence set $\tilde{Corr}=$ {(${\tilde{p}}_{i},{\tilde{q}}_{i}$) $\in R^{k\times 6}|i=1,2,\ldots ,$ k}, where ${\tilde{p}}_{i}\in \tilde{P}$ and ${\tilde{q}}_{i}\in \tilde{Q}$ denote the rectified coordinates. Finally, the optimal rigid transformation RT=[R,t] is computed via Singular Value Decomposition (SVD) based on $\tilde{Corr}$, recovering the best 3D registration between P and Q.

### 3.1. Problem Definition

Given a source point cloud P and a target point cloud Q, the goal of point cloud registration is to align the two point clouds via an unknown optimal 3D rigid transformation [R,t], comprising a rotation matrix R∈SO(3) and a translation vector $t\in R^{3}$. This transformation matrix can be obtained by minimizing the geometric error between the matched point pairs, as formalized in Equation (1):

(1) $$min\sum {\left\|R\cdot p_{i}+t-q_{i}\right\|}_{2}^{2},\;\left(p_{i},q_{i}\right)\in \vartheta ,i=1,\ldots ,N,$$

where ϑ denotes the set of ground-truth correspondences between P and Q, $p_{i}\in P,q_{i}\in Q$; N represents the number of points; and ${\left\|\cdot \right\|}_{2}^{2}$ refers to the squared Euclidean norm (L2 norm).

### 3.2. DCA Module (Dual-Consensus Attentive Feature Enhancer Module)

The Dual-Consensus Attentive Feature Enhancer (DCA) serves as the cornerstone of GeoCORNet for extracting robust and geometrically consistent features from low-overlap sensor point clouds. It directly addresses the challenges of noise, outliers, and sparse correspondences by synergistically enforcing dual geometric consistency constraints (distance and angular) and facilitating bidirectional contextual interaction between the source and target point clouds (representing distinct sensor viewpoints). The output features $\tilde{F_{P}}$ and $\tilde{F_{Q}}$ exhibit enhanced discriminative power within overlapping regions and suppressed responses elsewhere, providing a reliable foundation for subsequent correspondence estimation.

#### 3.2.1. Self-Attention Augmented Spatial Consistency

First, three multilayer perceptrons (MLPs) are employed to extract initial features $F_{P}$ and $F_{Q}$ from P and Q, respectively. These features are then enhanced with spatial consistency through dual local geometric constraints, including length consistency and angular consistency, yielding refined features $\bar{F_{P}}$ and $\bar{F_{Q}}$.

**Figure 2:**
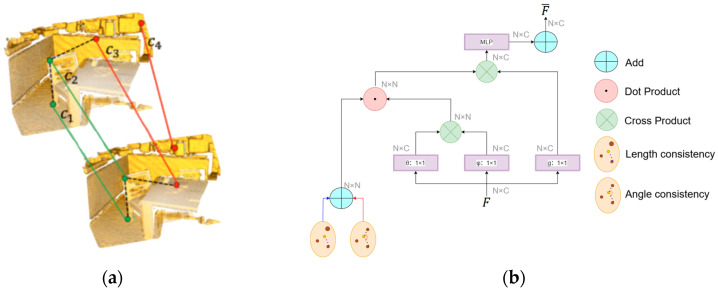
Spatial consistency illustration and implementation. (**a**) Spatial consistency. Points $c_{1},\;c_{2},\;c_{3},\;c_{4}\in P$, where $c_{1}$ was an inlier and $c_{3}$ was an outlier. Green lines indicated correct correspondences, while red lines denoted incorrect ones. The outlier $c_{3}$ shared the same distance to $c_{2}$ as the inlier $c_{1}$, but the neighborhood angle $∠c_{1}c_{2}c_{3}$ failed to maintain consistency after transformation. (**b**) Spatial consistency self-attention mechanism. Length (blue) and angular (red) consistency were fused with self-attention-based dynamic weighting to enhance geometric constraints on matched point pairs.

Specifically, length consistency is applied to enforce invariance of Euclidean distances between matched point pairs under rigid transformations, as formalized in Equation (2):

(2) $${\left\|p_{i}-p_{j}\right\|}_{2}\approx {\left\|R\cdot q_{i}+t-q_{j}\right\|}_{2}\;\;\;\;\;\;\forall \left(p_{i},q_{i}\right),\left(p_{j},q_{j}\right)\in \vartheta ,$$

where $p_{i}$ denotes two points in the overlapping region of the source point cloud P, and $q_{i}$ represents their corresponding points in Q. However, relying solely on distance constraints can overlook directional information. For instance, under anisotropic deformation or noise, point pairs with similar distances but divergent orientations might yield incorrect correspondences. As illustrated in Figure 2a, considering a neighborhood centered at $c_{2}$ while the outlier $c_{3}$ shares the same distance to $c_{2}$ as the true match $c_{1}$, their angular relationship (e.g., $∠c_{1}c_{2}c_{3}$) fails to remain consistent after transformation.

To solve ambiguities arising from distance-only constraints and enhance robustness to sensor noise and local deformations, we introduced spatial directional consistency constraints, proposing angular consistency to resolve false correspondences with similar length distances. This is achieved by computing the cosine similarity between feature vectors of point pairs, as formulated in Equation (3):

(3) $$cos\theta =\frac{f_{i}\cdot f_{j}}{\left\|f_{i}\right\|\left\|f_{j}\right\|},$$

where ${f_{i},f}_{j}$ represents the *i*-th and *j*-th points’ initial features of two points from P or Q. As demonstrated in Figure 2b, the fusion of length and angular consistency yields spatially enhanced features $\bar{F_{P}}$ and $\bar{F_{Q}}$, effectively eliminating directionally inconsistent spurious matches and overcoming the limitations of relying solely on distance constraints.

#### 3.2.2. Bidirectional Cross-Attention Mechanism

**Figure 3:**
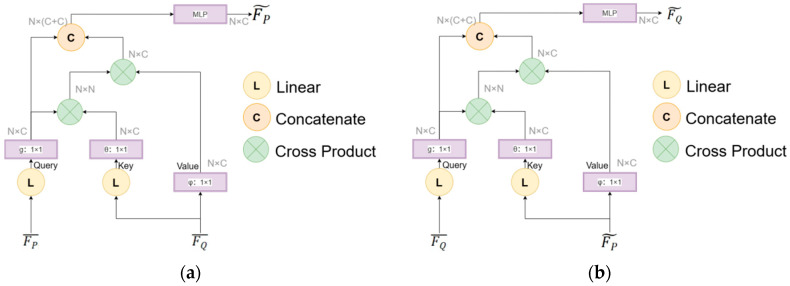
Bidirectional cross-attention mechanism. (**a**) Cross-attention from P to Q. (**b**) Cross-attention from Q to P.

A bidirectional cross-attention mechanism is introduced to establish global feature correlations between P and Q, dynamically capturing potential correspondences in low-overlap scenarios, as illustrated in Figure 3. This mechanism enables symmetric feature interaction between the two point clouds, mimicking the process of correlating information from multiple sensor acquisitions.

Specifically, we first computed cross-attention from P to Q. As shown in Figure 3a, the spatially enhanced feature matrix $\bar{F_{P}}$ is treated as Query, while $\bar{F_{Q}}$ serves as Key and Value. The attention weights are calculated using Equation (4):

(4) $$\alpha_{P\to Q}=Softmax\left(\frac{\bar{F_{P}}\cdot {\bar{F_{Q}}}^{T}}{\sqrt{d}}\right),$$

where d denoted the dimensionality of $\bar{F_{Q}}$. Based on the computed cross-cloud attention weights, $\bar{F_{P}}$ was aggregated via weighted summation as formulated in Equation (5):

(5) $$\tilde{F_{P}}=\bar{F_{P}}+\alpha_{P\to Q}\cdot \bar{F_{Q}},$$

Subsequently, cross-attention from Q to P is computed to refine feature representations bidirectionally. As illustrated in Figure 3b, $\bar{F_{Q}}$ acts as Query, while $\tilde{F_{P}}$ functions as Key and Value. The attention weights are derived via Equation (6):

(6) $$\alpha_{Q\to P}=Softmax\left(\frac{\bar{F_{Q}}\cdot {\tilde{F_{P}}}^{T}}{\sqrt{d}}\right),$$

where d represented the dimensionality of $\tilde{F_{P}}$. Using these weights, $\bar{F_{Q}}$ was updated through aggregation, as shown in Equation (7):

(7) $$\tilde{F_{Q}}=\bar{F_{Q}}+\alpha_{Q\to P}\cdot \tilde{F_{P}},$$

This bidirectional cross-attention mechanism generates weighted aggregated features $\tilde{F_{P}}\epsilon P$ and $\tilde{F_{Q}}\epsilon Q$, which fuses cross-cloud contextual information. The process enables adaptive focusing on overlapping regions while suppressing noise interference in non-overlapping areas.

### 3.3. Mutual-Confidence Correspondence Seeding (MCCS)

The MCCS module generates an initial correspondence set Corr by picking out top-k matched point-pairs as seed correspondences based on feature similarity, which serves as the confidence criterion. First, the feature similarity matrix S between P and Q is computed. Each element $s_{ij}\in S$, representing the confidence score of the point pair $\left(p_{i},q_{j}\right)$, is defined by Equation (8):

(8) $$s_{ij}=\frac{\tilde{f_{i}^{P}}\cdot \tilde{f_{j}^{Q}}}{\left\|\tilde{f_{i}^{P}}\right\|\left\|\tilde{f_{j}^{Q}}\right\|},$$

where $\tilde{f_{i}^{P}}\epsilon \tilde{F_{P}}$ and $\tilde{f_{j}^{Q}}\epsilon \tilde{F_{Q}}$ denoted the weighted aggregated features of points $p_{i}\in P$ and $q_{j}\in Q$, respectively.

To enhance reliability, a bidirectional nearest neighbor constraint was applied: only matches where $p_{i}$ and $q_{j}$ were mutually top-*k* nearest neighbors were retained. Specifically, $\left(p_{i},q_{j}\right)$ had to satisfy that $q_{j}$ was ranked among the top-*k* nearest neighbors of $p_{i}$ in Q and $p_{i}$ simultaneously was ranked among the top-*k* nearest neighbors of $q_{j}$ in P. The bidirectional nearest neighbor constraint reduced the initial number of matches from O(N×M) to O(k×k) (k≪N,M) by screening high-confidence matching point pairs, significantly reducing the computational load of displacement prediction in the PDR module.

### 3.4. Predictive Displacement Rectification Module (PDR Module)

**Figure 4:**
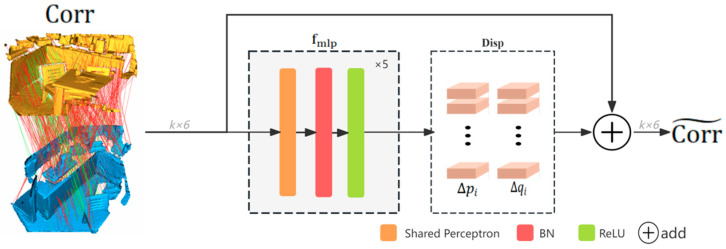
Architecture of the PDR module. The module takes the initial seed correspondence set Corr as input and employs a lightweight MLP-based displacement prediction network $f_{mlp}$ to predict offset vectors. These vectors form the set Disp = $\left\{(∆p_{i},∆q_{i})\in R^{k\times 6}|i=1,2,\ldots ,k\right\}$, which are then used to rectify erroneous correspondences in Corr.

The network architecture of our PDR module is illustrated in Figure 4. For the initial seed correspondence set Corr selected by the MCCS module, displacement vectors are computed to rectify erroneous matches, yielding the refined correspondence set $\tilde{Corr}=\{(\tilde{p_{i}},\tilde{q_{i}})\in R^{k\times 6}|i=1,2,\ldots ,k\}$, where each rectified pair $\left(\tilde{p_{i}},\tilde{q_{i}}\right)$ comprises the corrected 3D coordinates of the source and target points.

Specifically, the module takes the selected initial seed correspondence set Corr as input and utilizes a lightweight displacement prediction network (composed of multilayer perceptrons, MLPs) to estimate 3D displacement vectors $∆p_{i}\in R^{3}$ and $∆q_{i}\in R^{3}$ for each correspondence pair ($p_{i},q_{i}$), as formulated in Equations (9) and (10):

(9) $$\Delta p_{i}=f_{mlp}(p_{i},{p_{j})}_{j\epsilon N_{b}\left(i\right)},$$

(10) $$\Delta q_{i}=f_{mlp}(q_{i},{q_{j})}_{j\epsilon N_{b}\left(i\right)},$$

where $N_{b}(i)$ denoted the neighborhood of point i, containing b neighboring points. The predicted displacements are then applied to rectify the positions of the potentially erroneous sensor data points via Equations (11) and (12):

(11) $$\tilde{p_{i}}=p_{i}+∆p_{i},$$

(12) $$\tilde{q_{i}}=q_{i}+∆q_{i},$$

yielding geometrically coherent corrected correspondence set $\tilde{Corr}=\{(\tilde{p_{i}},\tilde{q_{i}})\in R^{k\times 6}|\;i=1,2,\ldots ,k\}$.

Residual connections, similar to ResNet blocks, are incorporated into the MLP to serve as the backbone for fine registration. Experiments shows that this design facilitates gradient propagation and improves training performance. The final regression layer is configured with 3 output channels to match the expected displacement dimensions. Outlier correspondences are corrected based on the predicted displacement vectors. The PDR module does not just filter sensor data but actively corrects and enhances its geometric plausibility, thereby enabling more robust and accurate registration crucial for reliable 3D sensing. The subsequent displacement loss function (Section 3.5.2) further ensures these corrected pairs conform to the expected distribution of inliers.

### 3.5. Loss Function

Our proposed network can achieve high accuracy and robustness in low-overlap point cloud registration through the joint optimization of Overlap Loss and Displacement Loss.

#### 3.5.1. Overlap Loss

The Overlap Loss is designed to constrain the model to accurately predict overlapping regions between point clouds, providing reliable priors for correspondence filtering. To address class imbalance in low-overlap scenarios, a Weighted Binary Cross-Entropy (BCE) Loss is employed, as formulated in Equation (13):

(13) $$L_{overlap}=-\frac{1}{N\cdot M}\sum_{i=1}^{N}\sum_{j=1}^{M}\left[\omega_{ij}\cdot {Μ}_{ij}\log\sigma \left(s_{ij}\right)+\left(1-{Μ}_{ij}\right)\log\sigma \left({1-s}_{ij}\right)\right],$$

where ${Μ}_{ij}$ denoted a binary overlap mask generated based on the ground-truth transformation $R_{gt}$ and $t_{gt}$, defined in Equation (14):

(14) $${Μ}_{ij}=\left\{\begin{array}{c} 1\;\;\;\;if\;{\left\|R_{gt}\cdot p_{i}+t_{gt}-q_{j}\right\|}_{2}<\delta \\ 0\;\;\;\;\;\;\;\;\;\;\;\;\;\;\;\;\;\;\;\;\;\;\;\;\;\;\;\;\;\;\;\;\;otherwise \end{array}\right.,$$

with δ representing the overlap threshold.

#### 3.5.2. Displacement Loss

To ensure the accuracy of the predicted displacement vectors and enforce corrected outliers to align with inlier distributions, a displacement loss function $L_{Disp}$ is designed, as formalized in Equation (15):

(15) $$L_{Disp}\left(\tilde{p_{i}},p_{t},\tilde{q_{i}},q_{t}\right)=min{\left\|\tilde{q_{i}}-q_{t}+{\left\|\tilde{p_{i}}-p_{t}\right\|}_{2}^{2}\right\|}_{2}^{2},$$

where $\left(\tilde{p_{i}},\;\tilde{q_{i}}\right)$ denoted the rectified correspondence pair, and $\left(p_{t},q_{t}\right)$ represented the nearest inlier pair for each $\left(\tilde{p_{i}},\;\tilde{q_{i}}\right)$. By minimizing this loss, the displacement vectors of outlier pairs were constrained to match the geometric distribution of inliers, ensuring robust correction.

Physically, $L_{Disp}$ minimizes the geometric deviation between rectified correspondences $\left(\tilde{p_{i}},\;\tilde{q_{i}}\right)$ and their nearest inliers $\left(p_{t},q_{t}\right)$. By pulling corrected outliers toward the inlier manifold, it enforces distributional alignment where sparse inliers define the valid transformation space. This contrasts with hard outlier rejection, which discards valuable geometric constraints.

#### 3.5.3. Total Loss

The total training loss is formulated as the weighted sum of the Overlap Loss and Displacement Loss, as defined in Equation (16):

(16) $$L_{Total}=\lambda_{o}L_{Overlap}+\lambda_{d}L_{Disp},$$

where the weighting coefficients were set to $\lambda_{o}=1.0$ and $\lambda_{d}=1.5$.

## 4. Results

In this section, comparative experiments were conducted between our network and other mainstream methods. Section 4.1 describes the experimental setup, including dataset selection, evaluation metrics, and implementation details. Section 4.2 depicts the comparative results of the registration performance for GeoCORNet and other methods on the benchmarks of 3DMatch and 3DLoMatch. Section 4.3 focuses on an ablation study to analyze the contributions of individual modules in GeoCORNet, while Section 4.4 investigates the impact of varying b values in PDR module. Section 4.5 quantifies computational efficiency and parameter complexity.

The proposed method was compared against five existing point cloud registration approaches: ICP [7], RANSAC [13], Predator [26], PointDSC [31], SC2-PCR [20], and PointTr [33]. Among these, ICP represent traditional registration methods, whereas Predator, PointDSC, SC2-PCR, and PointTr are learning-based approaches widely adopted in recent research.

### 4.1. Experiment Settings

#### 4.1.1. Datasets

Experiments were conducted on the benchmark datasets (3DMatch and 3DLoMatch) to evaluate the performance of GeoCORNet. 3DMatch includes 62 scenes captured from diverse sensors and complex layouts. Following standard protocols, 46 scenes were used for training, 8 for validation, and 8 for testing. The test set comprises 1623 partially overlapping point cloud fragments with their corresponding ground-truth transformation matrices. To specifically evaluate low-overlap scenarios, 3DLoMatch was constructed by picking out point-pairs from 3DMatch with overlap ratios below 30%, which served as a challenging benchmark for extreme low-overlap registration tasks.

#### 4.1.2. Evaluation Criteria

Three metrics were adopted to evaluate registration performance: Registration Recall (RR), Rotation Error (RE), and Translation Error (TE).

1. Registration Recall (RR)

The recall rate is calculated as follows:

(17) $$RR=\frac{1}{k}\sum_{i=1}^{k}\left[RMSE(T(\tilde{p_{i}}),\tilde{q_{i}})<\zeta \right],$$

where RMSE(·) denotes the root mean square error; *T*(·) is the ground-truth rigid transformation; $(\tilde{p_{i}},\tilde{q_{i}})\epsilon \tilde{Corr}$, ζ=0.1; and *k* represents the number of filtered correspondences.

2. Rotation Error (RE)

The angular deviation between predicted and ground-truth rotations is computed as follows:

(18) $$RE=arccos(\frac{Tr\left(R_{pred}R_{gt}^{T}\right)-1}{2}),$$

3. Translation Error (TE)

The Euclidean distance between predicted and ground-truth translations is defined as follows:

(19) $$TE={\left\|t_{pred}-t_{gt}\right\|}_{2},$$

Thresholds for rotation and translation errors across datasets are listed in Table 1.

#### 4.1.3. Implementation Details

In the preprocessing stage, input point clouds were voxelized with a voxel size of 0.05 m. From 3DMatch, 14,732 points were randomly sampled per point cloud, followed by voxel downsampling. GeoCORNet was implemented using PyTorch 1.7.1, and the Adam optimizer was employed for training. The initial learning rate was set to 0.001, decayed by 50% every 10 epochs, with a batch size of 32 and 300 training epochs on 3DMatch. For hyperparameters, the feature dimensionality was set to 128, and the parameter k in Section 3.3 was configured to 500. During training, the feature extraction module was pretrained first, followed by end-to-end training of the entire network using the pretrained features. Experiments were conducted on an Ubuntu 20.04 system with 32 GB of RAM and an NVIDIA RTX 3090 GPU (Nvidia, Santa Clara, CA, USA).

### 4.2. Evaluation on Indoor Scenes (3DMatch and 3DLoMatch)

To validate the performance of our method, comparative experiments were conducted against other mainstream point cloud registration approaches on large-scale benchmark datasets.

The quantitative comparison results of registration performance on both 3DMatch and 3DLoMatch for different methods are summarized in Table 2. As shown in the table, traditional methods (ICP) exhibited significant performance degradation in low-overlap scenarios, indicating their sensitivity to initial positions and inability to handle sparse correspondences, which highlighted their limitations under low-overlap conditions. On both 3DMatch and 3DLoMatch, the proposed GeoCORNet outperformed existing methods in the core registration metric, Registration Recall (RR), especially on 3DLoMatch, achieving 80.8% RR. On 3DLoMatch, although SC2-PCR slightly surpassed GeoCORNet in Translation Error (TE: 6.48 cm vs. 6.92 cm) on 3DLoMatch, our method achieved an 11.3% higher RR (80.8% vs. 69.5%) and a 23.8% lower Rotation Error (RE) (2.34° vs. 3.07°) compared to SC2-PCR, highlighting its robustness in low-overlap matching. While PointTr demonstrated marginally better RE and TE on 3DMatch (1.47°, 4.59 cm), its RR on 3DLoMatch (75.6%) was substantially lower than GeoCORNet (80.8%). This demonstrates that GeoCORNet can adapt to challenging scenarios (e.g., dynamic occlusions, low overlap) and provides more reliable solutions for practical applications.

Additionally, the robustness of registration method is validated by calculating the percentage of the differences in Registration Recall (RR), Rotation Error (RE), and Translation Error (TE) between the general-overlap scenarios (3DMatch) and low-overlap scenarios (3DLoMatch). The percentage of the differences are computed using Equation (20), where ${RR}_{H}$, ${RE}_{H}$, and ${TE}_{H}$ represent the Registration Recall, average Rotation Error, and average Translation Error on 3DMatch, respectively, while ${RR}_{L}$, ${RE}_{L}$, and ${TE}_{L}$ denote the corresponding metrics on 3DLoMatch.

(20) $$\begin{array}{c} RR↓=\frac{{RR}_{H}-{RR}_{L}}{{RR}_{H}}\times 100\%,\\ RE↑=\frac{{RE}_{L}-{RE}_{H}}{{RE}_{H}}\times 100\%,\\ TE↑=\frac{{TE}_{L}-{TE}_{H}}{{TE}_{H}}\times 100\%, \end{array}$$

The difference percentage results were presented in Table 3. As shown in the table, traditional methods (ICP) exhibited severe performance degradation in low-overlap scenarios, with RR decreases exceeding 60% and transformation errors nearly doubling, validating the limitations of traditional methods under low-overlap conditions. Among learning-based methods, although SC2-PCR slightly outperformed our method in translation error stability (TE↑ = 6.1% vs. 10.9%), its RR degradation (RR↓ = 25.3%) and rotation error increase (RE↑ = 50.5%) were 63.2% and 219.6% greatly higher than GeoCORNet (RR↓ = 15.5%, RE↑ = 15.8%), respectively, indicating significant degradation in registration success rate and rotational accuracy for SC2-PCR in low-overlap scenarios. While PointTr achieved better rotation and translation errors on 3DMatch (RE = 1.47°, TE = 4.59 cm), its RR↓, RE↑, and TE↑ (20.0%, 91.8%, and 23.9%) far exceeded GeoCORNet (15.5%, 15.8%, and 10.9%), particularly in rotation error degradation, revealing PointTr’s instability.

In contrast, the proposed GeoCORNet achieved the best performance in cross-dataset robustness metrics, with the smallest RR degradation (RR↓ = 15.5%) and rotation error increase (RE↑ = 15.8%), outperforming all existing methods. This validated the strong adaptability of our algorithm to scene variations. The results demonstrate that GeoCORNet possesses more reliable registration generalization capabilities and superior robustness.

**Figure 5:**
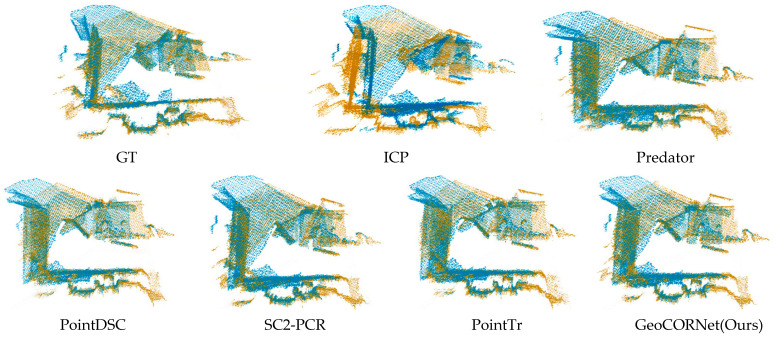
Comparison of registration performance across methods on the 3DMatch dataset. The source point cloud is colored in yellow and the target point cloud in blue. Ground truth (GT) alignment shown for reference. Our method achieves nearly perfect alignment with the ground truth (GT), while competing approaches (e.g., ICP, Predator, PointDSC, SC2-PCR, PointTr) exhibit visible misalignments in local regions.

**Figure 6:**
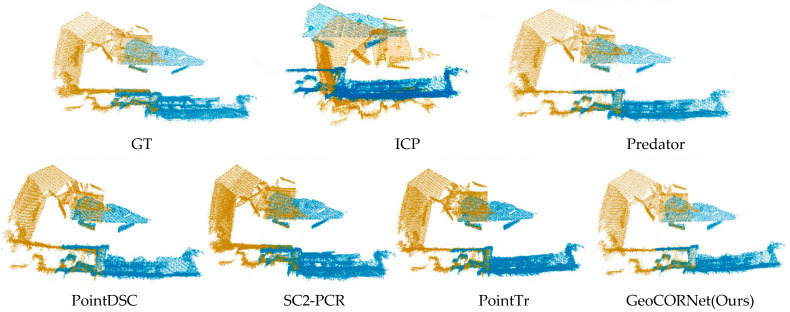
Comparison of registration results across methods on the 3DLoMatch dataset. The source point cloud is colored in yellow and the target point cloud in blue. Ground truth (GT) alignment shown for reference. GeoCORNet (ours) achieves the most accurate alignment with the ground truth (GT), while competing methods (e.g., ICP, Predator, PointDSC, SC2-PCR, PointTr) show significant angular errors in planar regions of the overlapping area.

The visualization results are illustrated in Figure 5 and Figure 6, where the source point cloud is colored in yellow and the target point cloud in blue.

Figure 5 shows the comparative registration results of traditional method (ICP) and five learning-based methods on 3DMatch. Our method achieved exceptional alignment accuracy, closely matching the ground truth (GT). It captured fine geometric details more precisely, with tighter edge alignment, whereas ICP, Predator, PointDSC, SC2-PCR, and PointTr exhibited local misalignment.

Figure 6 demonstrates the comparative registration results of traditional method (ICP) and five learning-based methods on 3DLoMatch. The registration results of GeoCORNet (ours) were the closest to GT. In the overlapping upper regions of the two point clouds, planar surfaces aligned almost perfectly by using our method, while other approaches introduced angular errors. These visual comparisons highlighted the superiority of our method in handling both standard and low-overlap scenarios.

### 4.3. Ablation Studies and Analysis

To investigate the importance of individual modules, an ablation study was conducted on 3DLoMatch. Specifically, the Baseline employed only a basic MLP architecture without any specialized modules. Subsequently, the DCA module, MCCS module, and PDR module were sequentially added to the Baseline. All experiments were performed under identical training strategies and hyperparameters to eliminate interference from other factors.

As shown in Table 4, the baseline model was highly sensitive to noise and outliers, resulting in a high mismatch rate. After incorporating the DCA module, geometric consistency in features was enhanced, enabling the attention of our network to be dynamically focused on overlapping regions. The DCA module accounted for approximately 54% of the total performance improvement, serving as the core driver. The MCCS module preliminarily filtered obvious outliers, reducing computational burden for subsequent modules; its contribution was relatively minor. The PDR module optimized correspondence positions, exhibiting high adaptability to occlusions and partial overlaps, and contributed 34% to the total improvement. Overall, our phased module design ensured controlled error propagation, ultimately achieving 80.8% RR on 3DLoMatch, a 42.1% improvement over the baseline.

Notably, the time cost progressively increases from 0.041 s (Baseline) to 0.130 s (Full) as modules are added, reflecting their computational demands: the DCA module dominates with 50% time share due to its O(N^2^) attention operations, while the MCCS and PDR modules contribute 15% and 19%, respectively. This demonstrates a favorable accuracy–efficiency trade-off, where the 3.7× time increase yields a 109% relative RR improvement.

### 4.4. b Value in Predictive Displacement Rectification and Analysis

In the PDR module, the parameter b denotes the number of local neighborhood points referenced during displacement prediction, which captures local geometric structures to optimize the accuracy of displacement estimation. Experiments were conducted on 3DLoMatch to evaluate the impact of b-values (5, 10, 20, or 30) based on Registration Recall (RR) and the mean positional error (TE). Parameters for the DCA module and MCCS module were kept consistent, with only the b-value in the PDR module adjusted. The following sections present the experimental results and analysis of how different b-values influence correction effectiveness.

As shown in Table 5, the optimal performance was achieved at b = 10, with RR = 80.8% and TE = 6.9 cm, balancing local geometric information and noise suppression. For b = 5, excessively small neighborhoods led to insufficient local geometric modeling (e.g., failure to capture surface continuity), increasing TE to 7.8 cm. For b = 20 and b = 30, overly large neighborhoods introduced noise from irrelevant regions (e.g., interference from distant points), degrading RR. Furthermore, when b-values were too large (e.g., 30), the likelihood of noise points participating in displacement prediction increased, leading to higher correction errors. Selecting an appropriate b-value (e.g., 10) could effectively filter isolated noise points while preserving local structural features.

In summary, smaller neighborhoods (b = 5) fail to capture surface continuity (e.g., planar regions), leading to unstable displacement predictions. Conversely, larger neighborhoods (b = 20/30) introduce irrelevant points from non-overlapping areas, increasing sensitivity to outliers. The value b = 10 empirically ensures sufficient local structural context while excluding distant noise points.

### 4.5. Complexity Analysis

To comprehensively evaluate practical applicability, which is critical for resource-constrained real-time applications, computational efficiency and model complexity are compared. The results measured on standardized hardware (Ubuntu 20.04 system with 32 GB of RAM and NVIDIA RTX 3090 GPU) are reported in Table 6.

As shown in Table 6, the proposed method achieved a better balance between computational efficiency and model complexity. GeoCORNet required only 0.13 s, which was significantly lower than the models with comparable parameter counts such as Predator (0.54 s) and PointTr (0.22 s). Although PointDSC (0.09 s) and SC2-PCR (0.09 s) exhibited faster inference speeds, their registration recall (RR) values were notably lower than that of GeoCORNet.

In summary, GeoCORNet achieves an optimal trade-off among computational efficiency (0.13 s), model complexity (3.8 M parameters), and registration accuracy (RR = 95.6%) through its parameter-shared lightweight design and joint optimization strategy.

### 4.6. Validation Under Extreme Low-Overlap Conditions

To address the critical challenge of point cloud registration under extremely sparse overlaps (e.g., in scenarios with severe occlusions or narrow sensor field-of-view), we conducted additional experiments on the 3DMatch dataset with overlap ratios below 10%. These test pairs were subsampled from the original 3DMatch test set by retaining only fragment pairs with overlap ratios strictly less than 10%.

As shown in Table 7, GeoCORNet achieves a Registration Recall (RR) of 62.3%, with an average Rotation Error (RE) of 5.62° and Translation Error (TE) of 11.83 cm. Compared to the 30% overlap scenario (80.8% RR, 2.34° RE, 6.92 cm TE), the performance degradation at 10% overlap is primarily attributed to the extreme sparsity of reliable correspondences, which compromises the effectiveness of both the DCA module and the PDR displacement rectification. Specifically, the scarcity of initial seed correspondences in the MCCS module (less than 20 pairs in some cases) reduces the geometric constraints available for transformation estimation, leading to higher sensitivity to noise and outliers.

## 5. Discussions and Limitations

As shown in Table 2 and Table 3, GeoCORNet achieved significant performance improvements in low-overlap point cloud registration. Specifically, the DCA module enhanced feature matching robustness in low-overlap scenarios by integrating distance consistency and angular consistency to establish dual-dimensional spatial geometric constraints. As illustrated in Figure 2a, traditional methods (e.g., ICP), which relied solely on Euclidean distance, struggled to distinguish false matches caused by local anisotropic noises. While existing deep learning methods introduced geometric self-attention, they failed to explicitly model spatial orientation. In contrast, as illustrated in Figure 2a, the DCA module suppressed erroneous matches with similar distances but inconsistent spatial orientations by fusing neighborhood angular relationships via cosine similarity. Additionally, the bidirectional cross-attention mechanism made our network dynamically focus on overlapping regions through symmetric feature interaction, providing more reliable initial correspondences. Consequently, the DCA module improved geometric consistency and initial matching reliability, as shown in Table 3 and Table 4, contributing 54% of the total performance gain. On 3DLoMatch, it boosted an RR by 22.5% compared to distance-only baselines.

Existing methods remove outliers via hard thresholds, which, while reducing noise interference, exacerbate information sparsity in low-overlap scenarios. The proposed correction strategy predicts displacement offsets using a lightweight MLP to rectify the outliers to the true distribution instead of discarding them. As shown in Table 2, Table 4 and Table 5, this module contributed 34% of the total performance improvement on 3DLoMatch, achieving a 4.11 cm reduction in TE compared to SC2-PCR. This result validated the dual advantages of our correction strategy: by retaining potentially useful information, it alleviated the insufficient correspondence problem caused by excessive outlier removal, while local neighborhood modeling enabled displacement prediction to capture geometric features such as surface continuity, avoiding interference from isolated noises. Through explicit geometric optimization, GeoCORNet achieved superior performance in Rotation Error (RE) metrics.

Traditional loss functions (e.g., mean squared error) struggle to address class imbalance in low-overlap scenarios. The proposed total loss function jointly optimizes overlap loss and displacement loss: the former enhances overlap region prediction through weighted cross-entropy, while the latter constrains the distribution of corrected point-pairs via geometric distance constraints. Data from Table 3 and Table 4 demonstrated that our design enables GeoCORNet to achieve a Registration Recall (RR) of 95.6% on 3DMatch, significantly surpassing baseline models relying on a single loss function. Compared to existing methods, the displacement loss explicitly strengthens spatial geometric consistency by minimizing the distance between corrected points and their nearest inliers, thereby achieving higher robustness in low-overlap scenarios.

Despite GeoCORNet’s strong performance in low-overlap registration, it still has limitations. In extreme low-overlap scenarios (e.g., overlap ratio < 10%), the extreme sparsity of matched point pairs may compromise the reliability of the displacement correction module. In such regimes, the sparsity of initial correspondences reduces the reliability of displacement rectification. As shown in Table 7, we tested on 3DMatches data with 10% overlap, observing an RR drop to 68.3% (vs. 80.8% at 30%). Future work will explore adaptive neighborhood selection and uncertainty-aware rectification to address this limitation.

Additionally, as shown in Table 5, the choice of neighborhood size b significantly impacts the effectiveness of displacement correction. Although experiments demonstrated optimal performance at b = 10, manual adjustment remains necessary across different scenarios, and future work needs to explore adaptive mechanisms for predicting b-values dynamically.

## 6. Conclusions

This paper presented GeoCORNet, a novel deep learning framework specifically designed to address the critical challenge of robust 3D point cloud registration under severe low-overlap conditions, a common limitation faced by LiDAR, depth cameras, and other 3D sensors in real-world applications like robotics and autonomous navigation.

GeoCORNet integrates key innovations for robust sensor data processing: the Dual-Consensus Attentive Feature Enhancer (DCA) enforces geometric consistency and employs cross-attention for reliable feature matching in overlapping regions. Crucially, the Predictive Displacement Rectification (PDR) module introduces a paradigm shift, moving beyond traditional outlier rejection to predictive correction of erroneous sensor correspondences. This maximizes the utility of sparse sensor data, transforming outliers into valuable inliers. Joint loss optimization further ensures geometric coherence.

Comprehensive evaluation on the 3DMatch and challenging 3DLoMatch sensor datasets demonstrated GeoCORNet’s superior performance. It achieved outstanding results, notably 80.8% Registration Recall (RR) and 2.34° Rotation Error (RE) on 3DLoMatch, showcasing exceptional robustness under low-overlap conditions.

These advances establish GeoCORNet as a powerful solution for enhancing the reliability and accuracy of 3D sensing systems. This work paves the way for more robust perception using ubiquitous sensors in complex, occlusion-prone environments.

## Figures and Tables

**Table 1:** Rotation and translation thresholds in different datasets.

Dataset	Rotation Threshold (°)	Translation Threshold (m)
3DMatch	15°	30 cm
3DLoMatch	20°	50 cm

**Table 2:** Quantitative comparison of registration performance across methods on 3DMatch and 3DLoMatch datasets. All best performances are highlighted exclusively through bold.

Method		3DMatch			3DLoMatch		
		RR (%)	RE (°)	TE (cm)	RR (%)	RE (°)	TE (cm)
Traditional	ICP [7]	86.6	3.16	9.67	18.3	8.25	25.41
Learning	Predator [26]	89.0	2.03	6.47	59.8	3.05	9.37
	PointDSC [31]	92.4	2.05	6.54	68.1	3.95	11.03
	SC2-PCR [20]	93.1	2.04	6.53	69.5	3.07	6.48
	PointTr [33]	94.5	**1.47**	**4.59**	75.6	2.82	**5.69**
	**GeoCORNet (Ours)**	**95.6**	2.02	6.24	**80.8**	**2.34**	6.92

**Table 3:** Cross-dataset robustness comparison across methods. ↑ = Improvement, ↓ = Reduction. All best performances are highlighted exclusively through bold.

Method		RR↓ (%)	RE↑ (%)	TE↑ (%)
Traditional	ICP [7]	78.9%	161.1%	162.8%
Learning	Predator [26]	32.8%	50.2%	44.8%
	PointDSC [31]	26.3%	92.7%	68.7%
	SC2-PCR [20]	25.3%	50.5%	**6.1%**
	PointTr [33]	20.0%	91.8%	23.9%
	**GeoCORNet** **(Ours)**	**15.5%**	**15.8%**	10.9%

**Table 4:** Ablation study on module contributions. baseline: utilized basic multilayer perceptrons (MLPs) for point cloud feature extraction, without attention mechanisms or geometric constraints. +DCA: added the DCA module to the baseline, introducing geometric consistency and bidirectional cross-attention. +MCCS: built upon +DCA, integrated the MCCS module to pick out high-confidence correspondences. +PDR: extended +MCCS with the PDR module for outlier rectification.

Module	3DLoMatch (RR↑)	RR Contribution Ratio	Time (s)
Baseline	38.7%		0.041
+DCA	61.2% (+22.5%)	54%	0.105 (+0.064)
+MCCS	66.4% (+5.2%)	12%	0.105 (+0)
+PDR	80.8% (+14.4%)	34%	0.130 (+0.025)

**Table 5:** Displacement prediction with varying neighborhood sizes (*b*-values). The parameter *b* (number of local neighborhood points) was tested at values 5, 10, 20, and 30 to analyze its impact on displacement prediction accuracy and registration performance. All best performances are highlighted exclusively through bold.

*b*-Value	RR (%)	TE (cm)
5	76.2	7.8
10	80.8	**6.9**
20	**81.5**	7.1
30	79.7	7.5

**Table 6:** Computational time and parameter count comparison of deep learning-based methods on the 3DMatch dataset. All best performances are highlighted exclusively through bold.

Method	Time (s)	Param. (M)	RR (%)
Predator	0.54	7.4	89.0
PointDSC	**0.09**	1.2	92.4
SC2-PCR	**0.09**	**0.9**	93.1
PointTr	0.22	7.6	94.5
**Ours**	0.13	3.8	**95.6**

**Table 7:** Quantitative comparison of registration performance at different overlap ratios on the 3DMatch dataset. All best performances are highlighted exclusively through bold.

Overlap Ratio	RR (%)	RE (°)	TE (cm)
10%	62.3	5.62	11.83
30%	**80.8**	**2.34**	**6.92**

## Data Availability

The 3DMatch dataset can be found at https://3dmatch.cs.princeton.edu/ (accessed on 6 July 2025).

## Abbreviations

The following abbreviations are used in this manuscript:

GeoCORNet	Geometric Consistency Optimization and Rectification Network
ICP	Iterative Closest Point
LiDAR	Light Detection and Ranging
MCCS	Mutual-Confidence Correspondence Seeding
MLP	Multi-layer Perceptron
PDR	Predictive Displacement Rectification
RANSAC	Random Sample Consensus
RE	Rotation Error
RR	Registration Recall
SLAM	Simultaneous Localization and Mapping
SVD	Singular Value Decomposition
TE	Translation Error
